# Increasing dietary soybean-derived trypsin inhibitor protein compromises nursery pig performance, nitrogen digestibility, and retention

**DOI:** 10.1093/jas/skaf253

**Published:** 2025-08-12

**Authors:** Mitchell J Nisley, Kayla A Miller, Joel D Spencer, Omarh F Mendoza, Hari B Krishnan, Nicholas K Gabler

**Affiliations:** Department of Animal Science, Iowa State University, Ames, IA 50011, USA; Department of Animal Science, Iowa State University, Ames, IA 50011, USA; United Animal Health, Sheridan, IN 46069, USA; The Maschhoffs LLC., Carlyle, IL 62231, USA; Agricultural Research Service, U.S. Department of Agriculture and Divisions of Plant Sciences and Animal Sciences, University of Missouri, Columbia, MO 65211, USA; Department of Animal Science, Iowa State University, Ames, IA 50011, USA

**Keywords:** nitrogen balance, nursery pig, trypsin inhibitor protein

## Abstract

Trypsin inhibitor proteins, intrinsic to soybeans and soy products, have been found to impair amino acid bioavailability, thereby attenuating pig growth performance. The objective of this experiment was to examine the effect of varying dietary trypsin inhibitor units (**TIU**) on the performance, nitrogen digestibility, and retention in nursery pigs. Sixty barrows (5.6 ± 0.65 kg body weight [**BW**]), weaned at 19 to 21 d of age, were randomly allocated across six dietary treatments (*n* = 10/treatment). Diets balanced for metabolizable energy (**ME**), crude protein, neutral detergent fiber (**NDF**), and standardized ileal digestible (**SID**) lysine were fed in two phases over the 42-d study, lasting 14 and 28 d, respectively. Dietary treatments were formulated on a soybean TIU protein (TIU/mg) basis, using soybean meal, raw whole soybeans, soybean oil, and soyhulls to target 0.5, 1, 2, 3, 4, and 6 TIU/mg in complete feed for both dietary phases. For phase 1 and 2 diets, analyzed TIU/mg were 0.22/0.54, 0.47/0.61, 1.71/1.98, 2.88/3.44, 3.79/4.01, and 6.15/5.42, respectively, averaging 0.38, 0.54, 1.85, 3.16, 3.9, and 5.79 TIU/mg across both phases. Pigs were individually housed with ad libitum access to water and feed. Pig BW and feed disappearance were measured at the start and end of each phase to calculate pig performance parameters. On day 17, a subgroup of 48 pigs (*n* = 8/treatment) were moved to metabolism crates for total fecal and urine collection to analyze nitrogen digestibility and retention. Data were analyzed using pig as the experimental unit to assess the effect of analyzed TIU levels, including linear and quadratic contrasts, on all parameters. No quadratic responses were reported overall (*P* > 0.05). At the end of the experiment, pig BW decreased from 25.3 to 15.5 kg as the diet increased from 0.38 to 5.79 TIU/mg (linear, *P* < 0.0001). Moreover, as TIU increased, overall average daily gain, average daily feed intake, and feed efficiency were attenuated, resulting in reductions by 49%, 32%, and 26%, respectively (linear, *P* < 0.01). During phase 2, at the highest levels of dietary TIU/mg, nitrogen digestibility (%) was reduced by 5.3% (linear, *P* = 0.001), and nitrogen retention (% of intake) decreased by 15.3% (linear, *P* < 0.0001). In conclusion, there was a constant reduction in growth performance, feed efficiency, and nitrogen retention as soybean TIU increased in the diet of nursery pigs.

## Teaser

Dietary trypsin inhibitor protein concentration above 0.54 TIU/mg results in a constant reduction in growth performance, feed efficiency, and nitrogen retention in nursery pigs.

## Introduction

Soybean co-products, such as soybean meal, remain essential feed ingredients to the swine industry due to their ability to provide a cost-effective source of amino acids (**AA**) in diet formulations. However, depending on processing method, soybean co-products contain varying levels of antinutritional factors, including trypsin inhibitor protein, phytate, lectin, and β-conglycinin (Heaney et al., 1991; Schulze et al., 1995; Krishnan et al., 2009; Gillman et al., 2015). Among these compounds, trypsin inhibitor proteins, in particular, have been shown to reduce nutrient digestibility in monogastric species (Barth et al., 1993; Gu et al., 2010; Kuenz et al., 2022). Functional trypsin inhibitor proteins competitively bind endogenous proteases, trypsin and chymotrypsin, consequently decreasing AA bioavailability by hindering proteolysis, potentiating pancreatic beta cell hypertrophy and hyperplasia (Melmed et al., 1976; Yen et al., 1977). Moreover, reduced AA digestibility potentiates hindgut proteolytic fermentation, which is often associated with increased diarrhea incidence in pigs (Zhang et al., 2020).

Soybean meal, the predominant soybean co-product used in swine diets, commonly contains trypsin inhibitor activities ranging from 2.0 to 22.0 trypsin inhibitor units (**TIU**) per mg (Kim and Krishnan, 2023). Generally, conventional processing of soybean meal post-oil extraction involves heat treatment, which has proven effective in denaturing, and thus inactivating most TIU, thereby attenuating their effects (Purushotham et al., 2007). Studies have demonstrated that treating soybean meal with heat, which subsequently lowered the TIU, improved both AA digestibility and feed efficiency in nursery pigs relative to feeding them untreated soybean meal (Zarkadas and Wiseman, 2005; Goebel and Stein, 2010). Furthermore, Miller et al. (2024) observed reductions in grower pig (~40 kg) body weight (**BW**) gain (9.3%) and apparent total tract digestibility (**ATTD**) of nitrogen (7.8%) when fed dietary TIU/mg of 9.38 compared to pigs fed a diet with 0.99 TIU/mg. Finally, Royer et al. (2022) suggested that a maximum of 2.0 TIU/mg in the total feed should be established, as exceeding this limit may reduce weight gain and feed efficiency in growing and finishing pigs.

Understanding the effect active TIU level has on nursery pig productivity will enable nutritionists to better understand how to best formulate soybean-derived ingredients in young pig diets. However, there are limited empirical data in nursery pigs that report dietary TIU impact on pig performance, nitrogen digestibility, and retention. Therefore, the objective of this research was to assess the impact varying levels of dietary TIU have on nursery pig performance, diarrhea incidence, nitrogen digestibility, and retention. It was hypothesized that increasing soybean-derived TIU in the diet of nursery pigs would subsequently decrease pig growth performance, nitrogen digestibility, and retention, in concordance with an increase in diarrhea incidence.

## Materials and Methods

All animal procedures were approved by the Iowa State University Institutional Animal Care and Use Committee (IACUC protocol #23-111).

### Animals, housing, and dietary treatments

Pigs were weaned at 19 to 21 d and transported ~5 h from a commercial sow farm to the Iowa State University Swine Nutrition Research Farm (Ames, IA). Sixty barrows (5.6 ± 0.65 kg BW, Camborough 1,050 × 337, [PIC, Hendersonville, TN]) were randomly assigned to one of six dietary treatments (*n* = 10/treatment) and individually housed across two rooms for the duration of the study. Each room had pens of identical size (122 cm × 42 cm × 61 cm) and had an identical layout. The rooms were maintained at a 12/12-h light/dark cycle, and were temperature-controlled within the thermoneutral zone of the pigs for the duration of the experiment. Meal form diets were fed in two phases, consisting of 14 and 28 d, respectively, over a 42-d nursery period.

The diet ingredients of raw soybeans and soybean meal were analyzed (**Table 1**) for moisture (method 930.15 of AOAC, 2007), ash (AOAC 942.05), crude protein (AOAC 990.03), NDF (AOAC 2001.11), acid detergent fiber based on Goering and Van Soest (1970), calcium and phosphorus (AOAC 985.01), crude fat (AOAC 2003.05), and crude fiber based on AOCS Ba 6a-05. The dispensable and indispensable AA composition of raw soybeans and soybean meal was determined by the Agricultural Experiment Station Chemical Laboratories at the University of Missouri–Columbia (Columbia, MO) by cation-exchange HPLC (L8900 Amino Acid Analyzer, Hitachi High-Technologies Corporation, Tokyo, Japan). Soybean ingredients and complete feed were analyzed for TIUs as previously described by Kim and Krishnan (2023) and were reported in **Tables 1–3**.

**Table 1.** Ingredient analysis of raw soybeans and soybean meal, as-fed

Parameter	Raw soybeans	Soybean meal
Trypsin inhibitor units, TIU/mg	57.38	2.99
Dry matter, %	88.96	88.15
Ash, %	5.72	6.62
Crude protein, %	34.60	46.50
Crude fat, %	19.50	1.59
Acid detergent fiber, %	6.20	7.40
Neutral detergent fiber, %	7.50	7.10
Calcium, total, %	0.29	0.62
Phosphorus, total, %	0.52	0.73
Indispensable amino acids, %		
Arginine	2.49	3.25
Histidine	0.90	1.27
Isoleucine	1.70	2.24
Leucine	2.63	3.53
Lysine	2.29	2.99
Methionine	0.50	0.61
Phenylalanine	1.74	2.34
Threonine	1.32	1.72
Tryptophan	0.50	0.79
Valine	1.73	2.32
Dispensable amino acids, %		
Alanine	1.47	1.96
Aspartic acid	3.88	5.13
Cysteine	0.53	0.62
Glutamic acid	6.08	8.26
Glycine	1.48	1.90
Hydroxyproline	0.07	0.06
Proline	1.76	2.24
Serine	1.48	1.77
Taurine	0.13	0.13
Tyrosine	1.34	1.61
Total amino acids	34.14	44.11

**Table 2.** Phase 1 diet composition, as-fed

	TIU/mg					
Ingredient, %	0.5	1.0	2.0	3.0	4.0	6.0
Corn, ground	48.41	40.55	40.99	40.70	40.56	39.51
Soybean meal, 46.5% crude protein	16.72	33.43	30.17	28.00	26.81	25.08
Whole raw soybean, ground	--	--	1.93	3.79	5.61	9.21
Hydrolyzed soy protein^1^	9.13	1.50	2.46	3.31	3.26	2.88
Whey, dried	10.42	10.41	10.42	10.42	10.42	10.42
Monocalcium phosphate, 21% P	1.30	1.13	1.13	1.13	1.13	1.13
Limestone	0.68	0.55	0.58	0.59	0.60	0.61
Salt	0.50	0.50	0.50	0.50	0.50	0.5
Soybean oil	1.50	1.90	1.61	1.36	1.09	0.67
Soybean hulls	1.37	0.98	0.91	0.92	0.74	0.72
L-lysine HCl	0.58	0.36	0.37	0.36	0.36	0.35
DL-methionine	0.29	0.25	0.24	0.24	0.24	0.24
L-threonine	0.24	0.17	0.17	0.16	0.16	0.16
L-tryptophan	0.05	--	--	--	--	--
L-valine	0.11	--	--	--	--	--
L-isoleucine	0.06	--	--	--	--	--
L-leucine	0.12	--	--	--	--	--
Phytase^2^	0.03	0.03	0.03	0.03	0.03	0.03
Oats groats	7.50	7.50	7.50	7.50	7.50	7.50
Zinc oxide, 72% Zn	0.39	0.39	0.39	0.39	0.39	0.39
Vitamin premix^3^	0.20	0.20	0.20	0.20	0.20	0.20
Trace mineral premix^4^	0.15	0.15	0.15	0.15	0.15	0.15
Carbadox^5^	0.25	0.25	0.25	0.25	0.25	0.25
Calculated nutrient composition						
Dry matter, %	90.53	90.10	90.13	90.15	90.13	90.09
ME^6^, Kcal/kg	3353	3353	3353	3353	3353	3353
SID^7^ Lysine, %	1.40	1.40	1.40	1.40	1.40	1.40
SID Lysine:ME, g/Mcal	4.17	4.17	4.17	4.17	4.17	4.17
SID amino acid:Lysine						
Lysine	1.00	1.00	1.00	1.00	1.00	1.00
Threonine	0.62	0.63	0.62	0.62	0.62	0.62
Methionine + Cysteine	0.58	0.59	0.58	0.58	0.58	0.58
Tryptophan	0.21	0.21	0.21	0.21	0.21	0.21
Isoleucine	0.59	0.64	0.63	0.63	0.63	0.63
Valine	0.66	0.68	0.66	0.67	0.66	0.67
Calcium:phosphorus	1.00	1.00	1.00	1.00	1.00	1.00
Calcium:available phosphorus	1.46	1.46	1.46	1.46	1.46	1.46
Crude protein, %	23.0	23.0	23.0	23.0	23.0	23.0
Neutral detergent fiber, %	6.6	6.6	6.6	6.6	6.6	6.6
Trypsin inhibitor units/mg	0.5	1.0	2.0	3.0	4.0	6.0
Analyzed composition						
Trypsin inhibitor units/mg	0.22	0.47	1.71	2.88	3.79	6.15

^1^HP 300, 56% crude protein, <1.3 mg/g trypsin inhibitor units; Hamlet Protein, Findlay, OH, USA.

^2^Optiphos 2500; Huvepharma, Peachtree City, GA, USA. Minimum activity of 250 phytase units (FTU)/kg complete feed.

^3^Vitamin premix provided 6,125 IU vitamin A, 700 IU vitamin D_3_, 50 IU vitamin E, 30 mg vitamin K, 0.05 mg vitamin B_12_, 11 mg riboflavin, 56 mg niacin, and 27 mg pantothenic acid per kilogram of diet.

^4^Trace mineral premix provided 22 mg Cu (as CuSO_4_), 220 mg Fe (as FeSO_4_), 0.4 mg I (as Ca(IO_3_)_2_), 52 mg Mn (as MnSO_4_), 220 mg Zn (as ZnSO_4_), and 0.4 mg Se (as Na_2_SeO_3_) per kilogram of diet.

^5^Phibro Animal Health, Teaneck, NJ, USA. Included at 50 g/ton.

^6^ME = Metabolizable energy.

^7^SID = Standardized ileal digestibility.

**Table 3.** Phase 2 diet composition, as-fed

	TIU/mg					
Ingredient, %	0.5	1.0	2.0	3.0	4.0	6.0
Corn, ground	64.09	63.71	63.30	62.75	62.19	61.12
Soybean meal, 46.5% crude protein	25.08	25.12	24.19	23.52	22.86	21.52
Whole raw soybean, ground	--	0.44	2.24	4.03	5.82	9.40
Hydrolyzed soy protein^1^	5.00	4.89	4.73	4.46	4.19	3.65
Monocalcium phosphate, 21% P	1.03	1.04	1.03	1.03	1.03	1.02
Limestone	0.74	0.74	0.74	0.74	0.75	0.75
Salt	0.50	0.50	0.50	0.50	0.50	0.50
Soybean oil	1.29	1.30	1.08	0.86	0.64	0.20
Soybean hulls	0.68	0.68	0.61	0.54	0.47	0.33
L-lysine HCl	0.52	0.51	0.51	0.50	0.49	0.48
DL-methionine	0.25	0.25	0.25	0.25	0.25	0.24
L-threonine	0.22	0.22	0.22	0.22	0.22	0.21
L-tryptophan	0.05	0.02	0.02	0.02	0.02	0.01
L-valine	0.07	0.06	0.06	0.06	0.06	0.06
L-isoleucine	0.03	0.03	0.03	0.03	0.02	0.02
Phytase^2^	0.03	0.03	0.03	0.03	0.03	0.03
Zinc oxide, 72% Zn	0.11	0.11	0.11	0.11	0.11	0.11
Vitamin premix^3^	0.16	0.20	0.20	0.20	0.20	0.20
Trace mineral premix^4^	0.15	0.15	0.15	0.15	0.15	0.15
Calculated nutrient composition						
Dry matter, %	89.43	89.42	89.40	89.37	89.34	89.29
ME^5^, Kcal/kg	3344	3344	3344	3344	3344	3344
SID^6^ Lysine	1.35	1.35	1.35	1.35	1.35	1.35
SID^6^ Lysine:ME, g/Mcal	4.03	4.03	4.03	4.03	4.03	4.03
SID amino acid:Lysine						
Lysine	1.00	1.00	1.00	1.00	1.00	1.00
Threonine	0.62	0.62	0.62	0.62	0.62	0.62
Methionine + Cysteine	0.58	0.58	0.58	0.58	0.58	0.58
Tryptophan	0.21	0.19	0.19	0.19	0.19	0.19
Isoleucine	0.59	0.59	0.59	0.59	0.59	0.59
Valine	0.67	0.67	0.67	0.67	0.67	0.67
Calcium:phosphorus	1.07	1.07	1.07	1.07	1.07	1.07
Calcium:available phosphorus	1.86	1.86	1.86	1.86	1.86	1.86
Crude protein, %	21.8	21.8	21.8	21.8	21.8	21.8
Neutral detergent fiber, %	7.3	7.3	7.3	7.3	7.3	7.3
Trypsin inhibitor units/mg	0.5	1.0	2.0	3.0	4.0	6.0
Analyzed composition						
Crude protein, %	18.1	18.5	19.1	18.3	20.5	19.5
Trypsin inhibitor units/mg	0.54	0.61	1.98	3.44	4.01	5.42

^1^HP 300, 56% crude protein; <1.3 mg/g Trypsin inhibitor units; Hamlet Protein, Findlay, OH, USA.

^2^Optiphos 2500; Huvepharma, Peachtree City, GA, USA. Minimum activity of 250 phytase units (FTU)/kg complete feed.

^3^Vitamin premix provided the following per kilogram of diet: vitamin A, 6,125 IU; vitamin D_3_, 700 IU; vitamin E, 50 IU; vitamin K, 30 mg; vitamin B_12_, 0.05 mg; riboflavin, 11 mg; niacin, 56 mg; and pantothenic acid, 27 mg.

^4^Trace mineral premix provided 22 mg Cu (as CuSO_4_), 220 mg Fe (as FeSO_4_), 0.4 mg I (as Ca(IO_3_)_2_), 52 mg Mn (as MnSO_4_), 220 mg Zn (as ZnSO_4_), and 0.4 mg Se (as Na_2_SeO_3_) per kilogram of diet.

^5^ME = Metabolizable energy.

^6^SID = Standardized ileal digestibility.

In both phases, diets were formulated to 0.5, 1, 2, 3, 4, and 6 TIU/mg, while being balanced for metabolizable energy, neutral detergent fiber (**NDF**), and standardized ileal digestible (SID) lysine, and essential SID AA ratios to lysine using soybean ingredients (**Tables 2 and 3**). Diets were formulated in accordance with the NRC (2012) nutrient recommendations for the size of the pig utilized. For phase 1 and 2 diets, analyzed TIU/mg were 0.22/0.54, 0.47/0.61, 1.71/1.98, 2.88/3.44, 3.79/4.01, and 6.15/5.42, respectively, averaging 0.38, 0.54, 1.85, 3.16, 3.9, and 5.79 TIU/mg across both phases.

### Pig performance and fecal consistency

Throughout the 42-d growth assay, all diets were provided ad libitum with water available via a water nipple. Pig BW and feed disappearance were recorded on days 0, 7, 14, 21, and 42, to calculate average daily gain (**ADG**), average daily feed intake (**ADFI**), and feed efficiency (**Gain:Feed; G:F**). These parameters are reported for each dietary phase as well as for the overall period. Pig fecal consistency scores were evaluated daily from days 0 to 14. Fecal consistency was assessed on a subjective ordinal scale ranging from 0 to 3: 0 indicated a solid, well-formed stool; 1 signified a soft, marginally formed stool; 2 represented a soft, non-formed stool; and a score of 3 indicated watery diarrhea. Pigs that failed to thrive were removed from the population at the discretion of the farm staff to avoid investigator bias, and removals and mortalities were recorded.

### Digestibility and nitrogen retention evaluation

On day 17, eight barrows per treatment (top 80th percentile ADG within each treatment) were selected and moved to metabolism crates and fed ~5% of their average BW of their respective phase 2 diet (**Table 3**). Pigs were allotted feedings twice daily at 0800 and 1600 h, with water provided ad libitum through a cup waterer. A 12-h light cycle was maintained, and the room temperature was kept between 25 °C and 31 °C. Following a 3-d adaptation period to the stainless-steel metabolism pens measuring 0.53 m × 0.71 m, total feces and urine were collected over a 4-d period. Urine collection buckets contained 8 mL 6N hydrochloric acid to prevent nitrogen volatilization and microbial growth. Urine and feces were collected twice daily and stored at −8 °C until further processed. At the end of the collection period, urine samples were weighed, homogenized, and subsampled for analysis. Total combined feces were dried at 75 °C, weighed, ground through a 2-mm screen, then mixed and subsampled before being stored in desiccators. Pigs were weighed after the collection period, and the total feed refusals were recorded.

Nitrogen content in feed, feces, and urine was analyzed using a thermo-combustion analyzer (TruMac N, LECO Corporation, St. Joesph, MO, USA). ATTD coefficients of nitrogen were calculated as previously described (Adeola, 2001). The nitrogen intake, nitrogen retained, and nitrogen retention coefficients were calculated:


$$\begin{array}{l} Nitrogen\;intake,\;g/d=\;\left(\left[\;feed\;intake\;\left(g/d\right)\;\times \;diet\;nitrogen\;\left(\%\right)\right]\;\times \;100\right) \end{array}$$

$$\begin{array}{l} Nitrogen\;retained,\;g/d=\left(nitrogen\;digested\;\left(g/d\right)-urine\;nitrogen\;\left(g/d\right)\right) \end{array}$$

$$\begin{array}{l} Nitrogen\;\frac{retained}{intake},\;\%=\;\left(\left[\frac{nitrogen\;retained\;\left(g/d\right)}{nitrogen\;intake\;\left(g/d\right)}\right]\;\times \;100\right) \end{array}$$

$$\begin{array}{l} Nitrogen\;\frac{retained}{digested},\;\%=\;\left(\left[\frac{nitrogen\;retained\;\left(g/d\right)}{nitrogen\;digested\;\left(g/d\right)}\right]\;\times \;100\right) \end{array}$$

### Statistical analysis

Data were analyzed using the GLIMMIX procedure of SAS v. 9.4 (SAS Institute, Cary, NC), respectively. All performance and nitrogen retention data were analyzed using a one-way ANOVA to examine the effect of dietary treatment (TIU/mg level), followed by Tukey’s HSD test for multiple comparisons. Linear and quadratic regression analyses were performed using the analyzed TIU levels as the predictor. Categorical fecal consistency score data were analyzed using ordinal logistic regression with categories 0, 1, 2, and 3. Bonferroni corrections were applied to account for multiple comparisons, and *P*-values were calculated through maximum-likelihood estimation using the Newton–Raphson optimization method. Fecal score data are presented as the relative frequency of pigs (%) that received scores of either “2” or “3”, which indicated diarrhea-like characteristics, for easier interpretation. Treatment combined mortality and removal rates were analyzed using a chi-square test. For all analyses, differences were considered significant when *P* < 0.05.

## Results

### Fecal consistency and general health

Overall, combined pig removal and mortality rates were not statistically different among treatments (*P* > 0.05). During the first 14 d, pigs consuming a diet with 0.47 TIU/mg exhibited higher diarrhea incidence compared to those fed a diet with 0.22 TIU/mg (*P *= 0.010; Table 4). However, this trend did not persist in pigs fed diets containing TIU/mg levels greater than 0.47. Although differences in diarrhea incidence were not observed in the first 7 d (*P* > 0.05), levels of dietary TIU/mg above the minimum level (0.22 TIU/mg), excluding pigs fed 3.79 TIU/mg, exacerbated diarrhea incidence in the second week (days 7 to 14; *P *= 0.001). By the end of the 42-d trial, pigs fed the highest level of dietary TIU manifested an unthrifty appearance, in concordance with slight lethargy and excessive hair growth.

**Table 4.** Effect of dietary trypsin inhibitor protein on fecal score consistency and livability

	TIU/mg^1^						
Parameter^2^	0.22	0.47	1.71	2.88	3.79	6.15	*P-*value^3^
Diarrhea incidence^4^, %							
Days 0 to 7	57.1	71.4	61.4	50.0	47.1	52.9	0.184
Days 7 to 14	18.3^c^	45.0^a^^,^^b^	43.3^a^^,^^b^	51.9^a^	23.7^b^^,^^c^	46.7^a^	0.001
Phase 1, days 0 to 14	39.2^b^^,^^c^^,^^d^	59.2^a^	53.1^a^^,^^b^	50.8^a^^,^^b^	36.2^d^	50.0^a^^,^^b^	0.010
Removals + mortalities, %	20	20	10	10	20	40	0.640

^1^Trypsin inhibitor units per mg of complete feed.

^2^Estimates represented by 9 to 10 observations per treatment.

^3^Diarrhea incidence rates were analyzed using a generalized linear model with an ordinal distribution. Removals and mortalities rates were analyzed using a chi-square test.

^4^Diarrhea incidence rate estimates are presented as the relative frequency of pigs (%) that received scores of either “2” or “3”, which indicated diarrhea-like characteristics.

^a,^

^b,^

^c,^

^d^Estimates lacking a common superscript are significantly different *P* < 0.05.

### Growth performance

The effects of TIU level on nursery pig performance are reported in **Table 5**. Pigs fed the highest TIU level (5.79 TIU/mg) experienced a 38.7% reduction in BW compared to pigs fed the lowest level (0.38 TIU/mg) by the end of the 42-d trial (15.5 vs. 25.3 kg, linear, *P* < 0.0001). The trend for attenuated pig BW with increased TIU level was observed as early as day 14 (linear, *P* = 0.033). Overall, pigs fed the highest level of dietary TIU experienced 48.9% and 26.4% reductions in ADG and feed efficiency, respectively, when compared to pigs fed the lowest level (linear, both *P* < 0.0001, respectively). Moreover, a decrease in ADFI was observed with increasing dietary TIU (linear, *P* = 0.002), most notably at the highest level (5.79 TIU/mg). During phase 1 (days 0 to 14), pigs fed increasing levels of dietary TIU experienced reductions of up to 54.5% in ADG and 49.4% in feed efficiency (linear; *P* = 0.016 and *P* < 0.0001, respectively). In phase 2 (days 14 to 42), pigs fed the highest level of TIU had depressed ADG and ADFI compared to pigs fed lower levels (both quadratic; *P* = 0.007 and *P* = 0.037, respectively). Moreover, pigs in phase 2 experienced a 25.7% reduction in feed efficiency if fed the maximal level of dietary TIU in the titration (linear, *P* = 0.0002).

**Table 5.** Effect of dietary trypsin inhibitor protein on nursery pig performance

	TIU/mg^1^								*P-*value^2^	
Parameter^3^	0.38	0.54	1.85	3.16	3.90	5.79	SEM	Trt	Linear	Quadratic
Body weights, kg										
d0	5.6	5.6	5.5	5.6	5.6	5.5	0.21	0.998	0.738	0.968
d7	6.6	6.2	6.3	5.8	5.9	5.7	0.40	0.591	0.097	0.691
d14	8.7	8.2	8.7	7.3	7.5	6.9	0.68	0.311	0.033	0.921
d21	11.5^a^	11.3^a^	10.9^a^^,^^b^	9.4^a^^,^^b^	9.4^a^^,^^b^	8.3^b^	0.69	0.012	0.0001	0.843
d42	25.3^a^	25.1^a^	25.0^a^	21.0^a^^,^^b^	22.0^a^^,^^b^	15.5^b^	1.38	0.0002	<0.0001	0.150
Phase 1, d0–14										
ADG, kg	0.22	0.18	0.23	0.13	0.14	0.10	0.040	0.141	0.016	0.890
ADFI, kg	0.28	0.25	0.29	0.21	0.23	0.20	0.035	0.438	0.079	0.896
G:F	0.85^a^	0.81^a^^,^^b^	0.80^a^^,^^b^	0.50^a^^,^^b^	0.52^a^^,^^b^	0.43^b^	0.092	0.003	<0.0001	0.314
Phase 2, d14–42										
ADG, kg	0.57^a^	0.57^a^	0.56^a^	0.49^a^	0.50^a^	0.30^b^	0.032	<0.0001	<0.0001	0.007
ADFI, kg	0.82^a^	0.75^a^^,^^b^	0.80^a^^,^^b^	0.76^a^^,^^b^	0.77^a^^,^^b^	0.58^b^	0.048	0.045	0.014	0.037
G:F	0.70^a^^,^^b^	0.79^a^	0.71^a^	0.65^a^^,^^b^	0.65^a^^,^^b^	0.52^b^	0.040	0.003	0.0002	0.268
Overall, d0–42										
ADG, kg	0.47^a^	0.46^a^	0.46^a^	0.37^a^^,^^b^	0.39^a^	0.24^b^	0.029	<0.0001	<0.0001	0.121
ADFI, kg	0.65^a^	0.60^a^^,^^b^	0.64^a^	0.58^a^^,^^b^	0.60^a^^,^^b^	0.44^b^	0.036	0.013	0.002	0.057
G:F	0.72^a^^,^^b^	0.80^a^	0.72^a^^,^^b^	0.64^b^^,^^c^	0.64^a^^,^^b^^,^^c^	0.53^c^	0.034	0.0006	<0.0001	0.642

^1^Trypsin inhibitor units per mg of complete feed averaged over phases. Analyzed complete feed TIU/mg per phase 1 and 2 were 00.22/0.54, 0.47/0.61, 1.71/1.98, 2.88/3.44, 3.79/4.01 and 6.15/5.42, respectively.

^2^ *P*-values are calculated with respect to treatment ANOVA and TIU/mg orthogonal polynomial contrasts.

^3^Estimates represented by 6-10 observations per treatment.

^a,^

^b,^

^c^Estimates lacking a common superscript are significantly different *P* < 0.05.

### Nitrogen digestibility and retention

The effects of dietary TIU on nitrogen digestibility, excretion, and retention are shown in **Table 6**. With increasing levels of dietary TIU, the ATTD nitrogen coefficients were reduced by as much as 5.3% (linear, *P *= 0.001). In addition, total nitrogen intake (g/d) and retention (g/d) were reduced in concordance as dietary TIU increased (linear, both *P *< 0.0001, respectively). Fecal, urine, and total nitrogen excretion (g/d) were not statistically different among dietary treatments (Treatment and linear *P* > 0.05, respectively). However, quadratic responses by dietary TIU were reported for urinary nitrogen excretion (*P* = 0.069) and total nitrogen excretion (*P* = 0.044), in which excretion was the highest for 3.44 and 4.01 TIU/mg treatments (**Table 6**). Notably, nitrogen retention as a percentage of intake and digested nitrogen decreased by up to 15.3% and 10.8%, respectively, as dietary TIU increased (linear; *P* < 0.0001 and *P* = 0.002, respectively).

**Table 6.** Effect of dietary trypsin inhibitor protein on nursery pig nitrogen digestibility and retention

	TIU/mg^1^								*P-*value^2^	
Parameter^3^	0.54	0.61	1.98	3.44	4.01	5.42	SEM	Trt	Linear	Quadratic
Nitrogen metabolism										
Nitrogen intake, g/d	16.1^a^	16.1^a^	15.6^a^^,^^b^	14.2^a^^,^^b^	14.8^a^^,^^b^	11.9^b^	0.93	0.022	<0.0001	0.226
Nitrogen ATTD^4^, %	89.4^a^^,^^b^	91.1^a^	88.2^a^^,^^b^	87.6^a^^,^^b^	87.6^a^^,^^b^	84.7^b^	1.31	0.034	0.001	0.689
Nitrogen excretion										
Fecal, g/d	1.7	1.4	1.8	1.8	1.8	1.8	0.21	0.795	0.360	0.509
Urine, g/d	2.0	2.2	2.3	2.8	2.7	2.2	0.27	0.281	0.191	0.069
Total, g/d	3.7	3.6	4.1	4.5	4.5	4.0	0.32	0.214	0.083	0.044
Nitrogen retention										
Retention, g/d	12.4^a^	12.4^a^	11.5^a^	9.7^a^^,^^b^	10.3^a^^,^^b^	7.9^b^	0.84	0.003	<0.0001	0.569
Retention, % of intake	77.2^a^	77.3^a^	72.5^a^^,^^b^	67.8^a^^,^^b^	68.3^a^^,^^b^	65.4^b^	2.64	0.007	<0.0001	0.482
Retention, % of digested	86.3	84.8	82.1	77.6	77.9	77.0	2.64	0.057	0.002	0.379

^1^Trypsin inhibitor units per mg of complete feed.

^2^ *P*-values are calculated with respect to treatment ANOVA and TIU/mg orthogonal polynomial contrasts.

^3^Estimates represented by 8 observations per treatment, d 20-24 of study.

^4^ATTD = apparent total tract digestibility.

^a,^

^b^Estimates lacking a common superscript are significantly different *P* < 0.05.

## Discussion

Soybean co-products such as soybean meal are widely fed to pigs throughout the world due to their ideal AA and energy profile. However, soybean meal and whole soybeans contain antinutritional factors, including trypsin inhibitor protein, phytate, lectin, and β-conglycinin (Heaney et al., 1991; Schulze et al., 1995; Krishnan et al., 2009; Gillman et al., 2015), that can impede pig growth performance and nutrient digestibility (Li et al., 1998; Zarkadas and Wiseman, 2005; Goebel and Stein, 2010). Of specific concern are trypsin inhibitor proteins that exert their antinutritional effect by acting as a substrate and binding endogenous proteases trypsin and chymotrypsin, inhibiting them from utilizing luminal peptides as a substrate (Melmed et al., 1976). As a result, the digestibility of AA ingested by the host is reduced, posing serious implications for animal nutrition. Nevertheless, there is limited work completed determining the level of dietary TIU in complete feed that is detrimental to nursery pig performance. This is an especially pertinent issue as newly weaned pigs lack the digestive capacity to efficiently digest proteins (Engelsmann et al., 2022). Therefore, the overall objective of the study herein was to assess the impact of varying levels of dietary TIU on nursery pig performance, diarrhea incidence, nitrogen digestibility, and retention.

Feeding elevated dietary TIU levels has been shown to decrease feed efficiency and weight gain in swine and other monogastric species (Yen et al., 1977; McNaughton, 1981; Kuenz et al., 2022). In the current study, pigs fed increasing levels of dietary TIU observed associated reductions in overall feed efficiency and weight gain, corroborating previous research. A linear reduction in pig BW was observed as early as day 14, exemplifying the adverse effect trypsin inhibitor proteins have on pig performance. Compared to the 0.38 TI/mg treatment, pigs fed the highest TIU level (5.79 TIU/mg) exhibited a 38.7% reduction in BW gain over the 42-d test period. Trypsin inhibitor proteins impair pig performance by directly reducing nutrient digestibility and feed efficiency, and indirectly limiting feed intake. This is evident during phase 1 (days 0 to 14), where feed intake among pigs across dietary treatments remained not different, but feed efficiency dropped by as much as 26.4% at the highest dietary TIU. The reductions in feed efficiency and weight gain present in phase 1 were maintained in phase 2 (days 14 to 42). Moreover, compared to the 0.50 TIU/mg treatment, feed intake decreased by as much as 29.3% at the highest dietary TIU, due to the lighter BW of pigs in the higher TIU treatment groups.

Monogastric species fed diets with elevated TIU levels have repeatedly shown diminished nitrogen digestibility (Barth et al., 1993; Schulze et al., 1995; Gu et al., 2010; Aderibigbe et al., 2021). Interestingly, a significant portion of the reduction in nitrogen digestibility may be due to endogenous secretions rather than exogenous excretions (Barth et al., 1993). In the current study, pigs fed the highest TIU level (5.42 TIU/mg) observed a 5.3% decrease in ATTD nitrogen compared to the lowest level (0.54 TIU/mg). Notably, the nitrogen retained as a percentage of intake and digested was 15.3 and 10.8%, respectively. The significant reduction in nitrogen retention as a percentage of digested at higher dietary TIU levels suggests a trypsin-manufactured AA imbalance, promoting increased nitrogen excretion in urine. Furthermore, fecal, urinary, and total nitrogen excretion (g/d) remained similar among dietary treatments. This observation can be explained by the decreased nitrogen intake associated with elevated dietary TIU levels, since total daily excretion (g/d) was not statistically different, yet proportionally much higher relative to intake.

Reduced nitrogen digestibility is a precursor to increased nitrogenous substrate entering the hindgut. Augmented proteolytic fermentation of peptides and AA in the hindgut of nursery pigs has been implicated to increase diarrhea incidence (Zhang et al., 2020; Xia et al., 2022), although there is limited literature associating trypsin inhibitor protein and diarrhea incidence. During the second phase of the current study, there was an increased incidence of diarrhea at every TIU level above the lowest level (0.22 TIU/mg), apart from 3.79 TIU/mg, which still exhibited a numerically higher prevalence. There were no observed differences in stool consistency during the first week, likely due to the stress of weaning exacerbating post-weaning diarrhea and masking any dietary effects (Moeser et al., 2017; Tang et al., 2022). Interestingly, pigs fed dietary TIU levels above the lowest level (0.22 TIU/mg) all observed similar diarrhea incidence, which may imply that the greater use of soybean meal, replacing hydrolyzed soy protein at the lowest level (0.22 TIU/mg), was the potential cause of increased diarrhea incidence.

It must be noted that the dietary TIU levels were modulated by the addition of raw soybeans and soybean meal. Therefore, dietary TIU concentrations essentially served as a proxy for raw soybean and soybean meal inclusion. Thus, the potentially confounding effects of other soybean inherent antinutritional factors could not be separated and elucidated when using raw whole soybeans. Previous research indicates that swine fed raw soybeans experience reduced weight gain in comparison to those fed diets consisting of soybean meal, wheat, or sorghum (Young, 1967; Knowles et al., 1995). Further research isolating potential antinutritional and nutritional factors that are intrinsic to soybeans is pivotal for genetic selection of soybeans and for optimizing their use as animal feed ingredients.

In this study, it was hypothesized that increasing soybean-derived TIU in the diet of nursery pigs would subsequently decrease pig growth performance, nitrogen digestibility, and retention, in concordance with an increase in diarrhea incidence. Herein, the first two null hypotheses were rejected, as nursery pigs fed higher soybean TIU protein levels experienced linear decreases in feed efficiency, weight gain, nitrogen digestibility, and retention. However, we could not reject the third null hypothesis, as increasing dietary TIU protein levels in nursery pig diets did not elicit an associated increase in diarrhea incidence. In conclusion, there was a constant reduction in growth performance, feed efficiency, and nitrogen retention as soybean TIU increased in the diet of nursery pigs. Trypsin inhibitor protein concentration should be monitored in soybean protein-containing feed and feed ingredients when fed to nursery pigs.

## Abbreviations

AA: amino acid
ADG: average daily gain
ADFI: average daily feed intake
ATTD: apparent total tract digestibility
BW: body weight
G:F: gain:feed
ME: metabolizable energy
NDF: neutral detergent fiber
SID: standardized ileal digestibility
TIU: trypsin inhibitor unit
